# P-750. The Use of Doxycycline for MRSA SSTIs in the Setting of Tetracycline Resistance (The DOXY-MASTER Study)

**DOI:** 10.1093/ofid/ofae631.946

**Published:** 2025-01-29

**Authors:** Ashly Nham, harminder sikand, Eva Sullivan

**Affiliations:** University of California, Davis Health, Sunnyvale, California; Scripps Mercy, Poway, California; Scripps Mercy San Diego, San Diego, California

## Abstract

**Background:**

Inducible doxycycline resistance has been reported in tetracycline (TET)-resistant methicillin-resistant *Staphylococcus aureus* (MRSA) isolates. The two major resistance mechanisms found in MRSA involve efflux pump expression and ribosomal protection proteins (RPP), both of which are mediated by the presence of *tet* genes. In isolates that are positive for *tetK* and negative for *tetM*, in vitro studies have demonstrated an increase in the mean minimum inhibitory concentration (MIC) of doxycycline above the Clinical and Laboratory Standards Institute (CLSI) breakpoint for susceptibility after incubation in broth containing doxycycline. This study aims to investigate the presence of inducible resistance as defined by treatment failure with the use of doxycycline for MRSA skin and soft tissue infections (SSTIs) in the setting of tetracycline resistance.

**Methods:**

This retrospective study analyzed patients from a 5-hospital health system and ensuing outpatient clinics from January 1, 2022 to February 29, 2024. Eligible patients were adults that had received doxycycline for the treatment of SSTI with a positive skin and soft tissue culture for MRSA that was susceptible to doxycycline. Exclusion criteria included presence of other organisms or concomitant infections requiring antimicrobial therapy, receipt of > 24 hours of effective systemic antimicrobial therapy against MRSA, and suspected or confirmed osteomyelitis or septic arthritis.

**Results:**

A total of 164 patients were screened, and 35 patients met the inclusion criteria for analysis: 11 in the TET-resistant arm and 24 in the TET-susceptible arm. Any readmission occurred in 6 (54.5%) patients in the TET-resistant arm and 7 (29.2%) patients in the TET-susceptible arm. However, readmission for SSTIs occurred in 1 patient (9.1%) in the TET-resistant arm and 2 patients (8.3%) in the TET-susceptible arm (p >0.999) with an average of 39.3 and 20.3 days to readmission (p = 0.179), respectively.

**Conclusion:**

In this small study sample, the use of doxycycline for MRSA SSTIs in the setting of tetracycline-resistance had no difference in treatment failure when compared to its use in tetracycline-susceptible isolates. This data suggests that susceptibility to doxycycline may be preserved in tetracycline-resistant MRSA isolates.

**Disclosures:**

**All Authors**: No reported disclosures

